# Probiotics in Irritable Bowel Syndrome: A Review Article

**DOI:** 10.7759/cureus.36565

**Published:** 2023-03-23

**Authors:** Shatakshi Sharma, Sunil Kumar, Sheeral Sajjad, Samriddhi Sharma

**Affiliations:** 1 Department of Medicine, Datta Meghe Institute of Higher Education and Research, Jawaharlal Nehru Medical College, Wardha, IND

**Keywords:** advantages of probiotics, treatment of ibs, diagnosis of ibs, probiotics rome, irritable bowel syndrome

## Abstract

Irritable bowel syndrome (IBS) is a persistent set of symptoms that reduces one's goodness of life. The treatment of these people is usually focused on reducing the symptoms caused by the condition. This article examines the function of probiotics in alleviating symptoms in IBS patients. The goal of studying the impact of probiotics on IBS patients is to research the changes they cause to the gut microbiota, which may be beneficial in preventing and treating such diseases over time. This article also discusses the pathophysiology, diagnostic standards, therapeutic modalities, probiotic sources, and therapeutic relevance for IBS patients.

## Introduction and background

The most frequent gastrointestinal functional disturbance is irritable bowel syndrome (IBS). It is a chronic illness that recurs periodically. Without an apparent structural or anatomical cause, this condition is characterised by stomach pain, bloating, and anomalies in bowel habits [1]. IBS symptoms change throughout time and are frequently accompanied by other functional gastrointestinal diseases and somatic pain disorders unrelated to the gastrointestinal tract [2]. Women are four times as likely as men to suffer from IBS. Age-related IBS symptom onset was predominately seen in individuals under 45. However, there is a higher prevalence in the later stages of life, among the elderly. Many instances start in childhood [1]. Based on how the ailment appears, IBS is currently categorised into four subtypes: the IBS-C subtype, the IBS-M subtype, unclassifiable IBS, and variants that are mostly constipatory.

**Figure 1:**
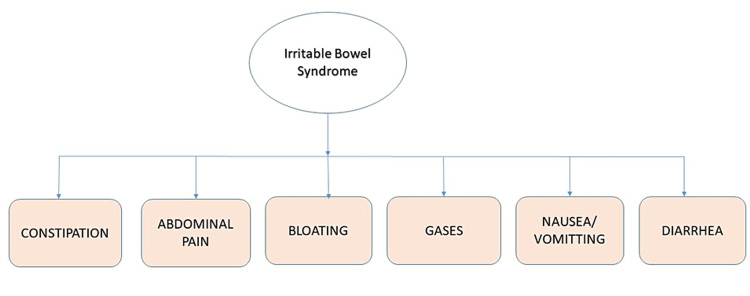
Symptoms of irritable bowel syndrome Adapted from source [1-5].

IBS sufferers may experience stomach pain, discomfort, flatulence, nausea, dyspepsia, reflux, and changing bowel patterns [3]. IBS's cause is still a mystery. However, various causes may have contributed to the disease's cause. The gut-brain axis, or the bidirectional communication pathways between the gut, its bacteria, and the central nervous system, may be compromised in addition to psychological issues by altered intestinal motility, food hypersensitivity, genetics, abnormalities of the intestinal microbiota, and other factors [4]. Furthermore, alterations caused by infection or bacterial overgrowth in the digestive system are thought to be IBS initiators. Childhood physical or sexual abuse, a brief breastfeeding period, any food allergies, being overweight, or any surgical procedures may also impact the development of the illness. IBS patients are more prone to have psychological disorders like anxiety or depression. Therefore, psychological counselling is advised for these people in place of or in addition to medicine [3]. The most popular drugs for treating IBS are laxatives, antispasmodics, and antidiarrheal medications, but prolonged usage can have serious adverse effects [5]. Instead of using these elements separately, synbiotics mix probiotics and prebiotics to improve the host's health. There are two types of synbiotics: complementary, which affect the host's natural microbiota, and synergistic, which use prebiotics as a substrate for provided probiotics [3]. Figure 1 shows symptoms of IBS.

## Review

Methodology

**Figure 2:**
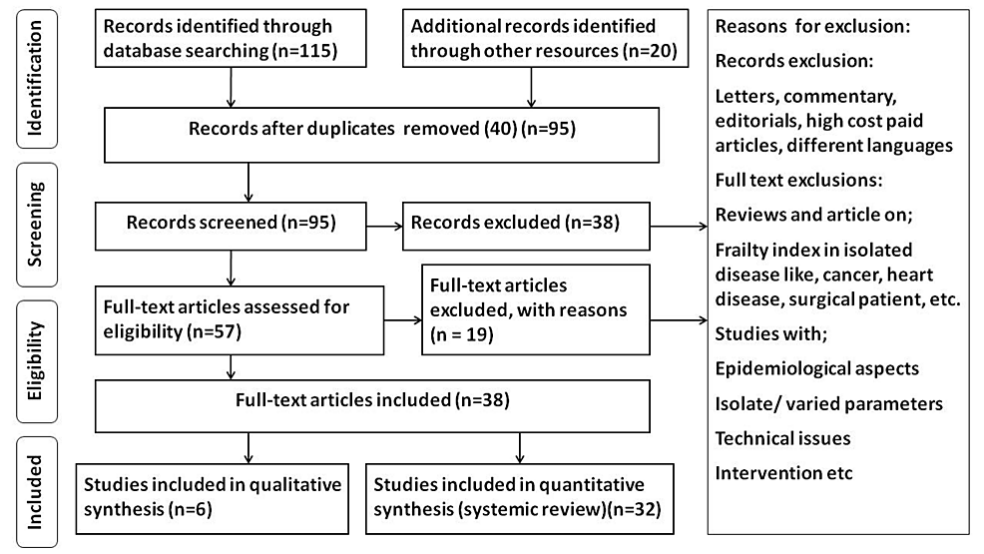
PRISMA model for the search strategy

We used PubMed to search Medline and the Cochrane Library to search CENTRAL databases. PubMed's search method was adapted to particular databases and was as ("irritable bowel syndrome"[Title/Abstract]) AND ("Rome criteria"[Title/Abstract])) OR ("probiotics"[Title/Abstract])) OR ("IBS treatment"[Title/Abstract])) OR (" advantages of probiotics"[Title/Abstract]). In addition, we searched the references list for potentially relevant papers for further investigations. Studies found through these electronic searches and relevant sources included in their bibliographies were examined. Original studies in English that assessed the risk factors, diagnosis, and management were included. Figure 2 depicts the PRISMA research approach.

Etiopathogenesis

The probable contributors are described below.

Genetic Predisposition

Having a parent with IBS is a higher predictor of acquiring the disorder than having a twin, indicating that environmental factors may be more critical than hereditary ones [6].

Intestinal Hypersensitivity

Because of this selective hypersensitization, which may cause IBS symptoms, different visceral afferent nerve fibre receptors in the gut wall are stimulated due to bowel distention or bloating. Instead of increasing neurosensory sensitivity, the colon's heightened sensitivity may be caused by a psychological propensity to report discomfort and urgency [7].

Psychopathology

Psychological concerns can disrupt the brain-gut axis by increasing the release of corticotropin-releasing hormone, which influences mood, digestive motility, visceral sensitivity, and inflammatory pathways via neuroendocrine and autonomic outputs. Stress activates the hypothalamic-pituitary-adrenal axis in IBS patients. As a result, cortisol levels, adreno-corticotropic hormone (ACCH), and other pro-inflammatory interleukins rise [8].

Altered Gut Microbiota

Compared to healthy people, IBS patients have lower faecal microbial diversity and different gut flora, suggesting a causal involvement in developing and maintaining IBS. Patients suffering from IBS were shown to have lower levels of lactobacilli and bifidobacteria, and their activities were severely hindered. There is proof that probiotics can normalize the interaction between pro- and anti-inflammatory cytokines via stabilizing microbiota and affecting intestinal fermentation. These findings positively impact visceral sensitivity, intestinal permeability, and inflammation [1].

Food Intolerance

In recent years, it has been shown that people without celiac disease have gastrointestinal pain and symptoms similar to IBS after consuming gluten. Like other well-known causes, gluten most likely affects intestinal permeability, causing the usual IBS symptoms by activating the enteric and autonomous neural systems [1].

Diagnosis of IBS

The patient should describe their pain or discomfort in detail, including its time, intensity, location, and whether it is generalized abdominal pain. IBS patients may have any of four bowel patterns, which are classified under Rome IV as diarrhoea predominant (IBS-D), constipation predominant (IBS-C), mixed diarrhoea and constipation (IBS-M), or unclassified (IBS-U). According to the Rome IV IBS Diagnosis Criteria, there must be at least two of the following symptoms in addition to stomach discomfort that comes and goes at least once per week in the last three months: urination-related or diagnostic symptoms, including those with a change in the appearance or frequency of faeces [9]. Immunology, biochemical and inflammatory markers, and blood counts should all be tested for celiac disease. When additional risk factors or concerning symptoms are present, the choice to do more research is made.

Conventional IBD treatment

The objective is to improve patients' quality of life while reducing the likelihood of complications and invasive procedures [10]. IBS can be treated with mesalazine, corticosteroids, immunosuppressive drugs, and monoclonal antibodies that target tumour necrosis factor (TNF) [11], but they have potential side effects. A new study is being done on treating those afflicted using corticosteroids, biosimilars, transforming growth factor (TGF)-beta, immunomodulators, anti-TNF medicines, and even altering the gut flora [12]. Ciprofloxacin, metronidazole, rifaximin, clarithromycin, and antituberculosis regimens are typically used in clinical studies with antibiotic treatment, either in combination or not with steroids or immunosuppressants [13]. Due to adverse effects, individuals with IBD have increasingly sought non-pharmacological therapies, such as cognitive behaviour therapy, hypnotherapy, psychotherapy, meditation, yoga, acupuncture, and exercise [14]. Probiotics still have a lot of contradictory research in that setting, but they are emerging as a fresh approach to treating these disorders [15].

Physiological Advantages of Probiotics in IBS

Probiotics contain a single or mixed culture of live bacteria that have been shown to improve health by modifying gut flora [16]. IBS patients can benefit from probiotics by having less bloating, discomfort, and frequent bowel movements. On the mucous membrane surface, they have antibacterial, antiviral, and anti-inflammatory characteristics that can slow or stop the progression of post-infective IBS. Probiotics are promising for symptom relief [17]. Probiotics are live microorganisms with several therapeutic applications for gastrointestinal illnesses. They are being investigated and used to treat gastrointestinal conditions, including Clostridium, pouchitis, antibiotic-associated diarrhoea, colitis, inflammatory bowel disease, and IBS [18]. Probiotics benefit the intestinal mucosa via numerous hypothesized processes, including reducing harmful bacteria growth and binding, enhancing epithelial barrier function, and modulating the host immunological response [19,20]. Short-chain fatty acids released by probiotics reduce luminal pH and boost the production of bactericidal proteins [20]. Butyric acid, a byproduct of fibre fermentation, has been shown to nourish colonic enterocytes and promote mucosal integrity [21]. DNA from probiotic organisms has also prevented the death of epithelial cells [22]. Numerous studies have demonstrated how probiotic bacteria can affect the human gastrointestinal (GI) mucosa's immune system functions [23]. Probiotics boost resistant cell populations in IBS, and immunomodulation is an essential component of their mechanism of action. Specific probiotic bacteria appear to have a direct effect on stomach pain. *Lactobacillus acidophilus* boosted the expression of m-opioid and cannabinoid receptors in colonic epithelial cell lines [24]. Pretreatment with the probiotic reduced pain in a rat stress model of visceral hypersensitivity. In a rat model of antibiotic-induced visceral hypersensitivity, Lactobacillus paracasei reduces gastrointestinal pain and mucosal inflammation similarly [25]. Probiotics have also been proven to affect the GI mucosa's integrity [26], which improves barrier protection [27]. Surprisingly, the sucralose urine excretion test resulted in no change in the colonic permeability, suggesting that the effects are restricted to the small bowel. Many studies have revealed that people with IBS have higher GI permeability [28,29]. Through this mechanism, therapies that enhance barrier function may be able to lessen symptoms. However, our understanding of the specific mechanisms of probiotic microbes is limited. The findings indicate that these effects are often strain- or species-specific. As a result, generalizing data from one probiotic to another is not advised.

Probiotics Sources

Kefir, curd, and other fermented foods are familiar sources of probiotics. They include various microorganisms that may improve gut health: delbrueckii subspecies, Thermophilus, and Bacillus strains. Bifidobacterium strains and Bulgaricus are the most widely employed organisms in these sources. According to research, they improve gut health and general anti-inflammatory and immunological responses [30]. In addition to the normal bacteria and Bifidobacterium species, Enterococcus and Streptococcus species are widely utilized in probiotic formulations. Several probiotic product formulations are available, ranging from bacteria to yeast species and even *Aspergillus oryzae*, a fungus of the filamentous type; depending on practicality, all of these may be made available as tablets, powders, capsules, pastes, sprays, or capsules. Probiotic use is a more natural alternative to pharmaceutical treatments with fewer side effects [30].

Prognostic and Therapeutic Importance of Probiotics

Probiotics have long been used to treat gastrointestinal issues, including traveller's diarrhoea, rotavirus enteritis, IBDs, bowel cancer, acute pancreatitis, aids diarrhoea, and IBS [31]. Probiotics in the colonic mucosa have been proven in studies to aid in the production of essential nutrients, the removal of toxins, the improvement of intestinal immunity, the prevention of microbial translocation, and the healing of a disrupted gut mucosal barrier [32]. The advantages of probiotic strains include greater stool consistency, a reduction in non-specific symptoms in healthy individuals, and benefits in inflammatory gastrointestinal diseases. Therefore, probiotics produce beneficial effects in the gut when ingested in appropriate proportions [33]. The investigation by Dimidi et al. showed that people with painful constipation had lower levels of Lactobacilli and Bifidobacterium and higher levels of breath methane. Specific probiotic strains can influence gastrointestinal activities such as secretion and motility, altering the local environment of the lumen [34]. To assess the efficiency of commercially available probiotics, a combination probiotic pill containing bacterial strains of eight distinct kinds: three strains of Bifidobacterium, four strains of Lactobacillus bacteria, and one strain of Streptococcus, was administered to individuals with IBS. According to Kim et al., individuals with bloating and IBS underwent randomized, double-blind studies for four to eight weeks of treatment. In the same individuals, the study results highlighted decreased flatulence and slowed colonic transit without affecting stool function. The study found that respondents did not experience sufficient alleviation from bloating, stomach discomfort, and stool-related symptoms [35]. In a comparable group of patients throughout eight weeks, Dolin conducted a randomized, double-blind, placebo-controlled clinical investigation to assess the advantages of a probiotic containing *Bacillus coagulans*. Patients were given *B. coagulans* or a placebo.

Compared to the placebo group, the study had a favourable effect by significantly decreasing the daily average of bowel movements [36]. In their study, Sun et al. looked at *Clostridium butyricum*'s efficacy in treating IBS-D. A four-week, randomized, double-blind, prospective, multicentric, and placebo-controlled research with *C. butyricum* involved a group of patients. It has been demonstrated that the probiotics of this species of the Clostridium genus improve the overall symptoms, frequency of stools, and quality of life in IBS-D patients [37].

As a result, the predictive function of probiotics in IBS must be further explored because, given the chronic nature of the illness, they may have several potential benefits. Still, pharmaceutical therapy for the same group may result in various adverse effects over time. Acknowledging the significant advantages of probiotics in IBS and encouraging consumers to benefit from their wealth of advantages is crucial. Finding specific strains of probiotics that reduce symptoms in IBS patients should be the primary goal of future research [38].

Limitations

Given the pathophysiology of IBS, establishing a verified aetiology of the condition as of today is beyond the scope of this article. Furthermore, we analyzed papers published throughout these years on only some strains in probiotic combinations. As a result, the adequacy of different species or mixes of species cannot be differentiated. While ignoring the importance of lifestyle changes and other relevant treatment modalities in quicker recovery of long-term IBS symptoms, this article has restricted its discussion to probiotics in IBS [38].

## Conclusions

It is worth mentioning that including probiotics in IBS patients' daily routines resulted in significant relief. A range of probiotic strains have been demonstrated to be helpful. Consequently, we may conclude that probiotics are advantageous for long-standing conditions like IBS. Prospective studies include a call for researchers to participate in studies that might lay out the treatment possibilities for those with IBS by combining some of the strains, evaluating their competitiveness in the intestinal environment, and keeping in mind their advantages. Using strain-specific probiotics for symptomatic treatment in IBS patients would significantly advance gastroenterology. Studies have shown an alleviation of symptoms, including bloating and stomach pain, and an overall improvement in IBS symptoms. The research utilising many probiotic supplement strains showed a favourable tendency for probiotic supplementation, but the trials using a single strain did not. A preliminary study indicates oral probiotic treatment may help reduce intestinal inflammation in cases of animal colitis in clinical trials. Because probiotic usage varies in the following ways, the effects of probiotic therapy frequently change in each clinical study: dosage, frequency, and length of use. Our knowledge of probiotics' structure and functionality is growing due to recent investigations. Oral probiotics can enhance several layers of intestinal defence and normalise the features of aberrant native microflora. On the other hand, when normalised in the inflamed mucosa of IBD patients or healthy individuals, many probiotic bacteria may have various immunological effects and specific features. In this regard, it may be actively investigated what different probiotic strains can do to locate and screen the most suitable components for the therapeutic intervention of IBD.

## References

[1] Radovanovic-Dinic B, Tesic-Rajkovic S, Grgov S, Petrovic G, Zivkovic V (2018). Irritable bowel syndrome - from etiopathogenesis to therapy. Biomed Pap Med Fac Univ Palacky Olomouc Czech Repub.

[2] Noddin L, Callahan M, Lacy BE (2005). Irritable bowel syndrome and functional dyspepsia: different diseases or a single disorder with different manifestations?. MedGenMed.

[3] Chlebicz-Wójcik A, Śliżewska K (2021). Probiotics, prebiotics, and synbiotics in the irritable bowel syndrome treatment: a review. Biomolecules.

[4] Van Malderen K, De Winter BY, De Man JG, De Schepper HU, Lamote K (2020). Volatomics in inflammatory bowel disease and irritable bowel syndrome. EBioMedicine.

[5] Su XT, Wang LQ, Zhang N (2021). Standardizing and optimizing acupuncture treatment for irritable bowel syndrome: A Delphi expert consensus study. Integr Med Res.

[6] Bellini M, Gambaccini D, Stasi C, Urbano MT, Marchi S, Usai-Satta P (2014). Irritable bowel syndrome: a disease still searching for pathogenesis, diagnosis and therapy. World J Gastroenterol.

[7] Nozu T, Kudaira M, Kitamori S, Uehara A (2006). Repetitive rectal painful distention induces rectal hypersensitivity in patients with irritable bowel syndrome. J Gastroenterol.

[8] Stasi C, Rosselli M, Bellini M, Laffi G, Milani S (2012). Altered neuro-endocrine-immune pathways in the irritable bowel syndrome: the top-down and the bottom-up model. J Gastroenterol.

[9] Schmulson MJ, Drossman DA (2017). What is new in Rome IV. J Neurogastroenterol Motil.

[10] Gomollón F, Dignass A, Annese V (2017). 3rd European evidence-based consensus on the diagnosis and management of Crohn’s disease 2016: Part 1: diagnosis and medical management. J Crohns Colitis.

[11] Harbord M, Eliakim R, Bettenworth D (2017). Third European evidence-based consensus on diagnosis and management of ulcerative colitis. Part 2: current management. J Crohns Colitis.

[12] Weisshof R, El Jurdi K, Zmeter N, Rubin DT (2018). Emerging therapies for inflammatory bowel disease. Adv Ther.

[13] Nitzan O, Elias M, Peretz A, Saliba W (2016). Role of antibiotics for treatment of inflammatory bowel disease. World J Gastroenterol.

[14] Torres J, Ellul P, Langhorst J (2019). European Crohn’s and Colitis Organisation topical review on complementary medicine and psychotherapy in inflammatory bowel disease. J Crohns Colitis.

[15] Marchesi JR, Adams DH, Fava F (2016). The gut microbiota and host health: a new clinical frontier. Gut.

[16] Hadley SK, Gaarder SM (2005). Treatment of irritable bowel syndrome. Am Fam Physician.

[17] Ford AC, Talley NJ (2012). Irritable bowel syndrome. BMJ.

[18] Aragon G, Graham DB, Borum M, Doman DB (2010). Probiotic therapy for irritable bowel syndrome. Gastroenterol Hepatol (N Y).

[19] Collins SM (2002). A case for an immunological basis for irritable bowel syndrome. Gastroenterology.

[20] O'Mahony L, McCarthy J, Kelly P (2005). Lactobacillus and bifidobacterium in irritable bowel syndrome: symptom responses and relationship to cytokine profiles. Gastroenterology.

[21] Moayyedi P, Ford AC, Talley NJ, Cremonini F, Foxx-Orenstein AE, Brandt LJ, Quigley EM (2010). The efficacy of probiotics in the treatment of irritable bowel syndrome: a systematic review. Gut.

[22] Niv E, Naftali T, Hallak R, Vaisman N (2005). The efficacy of Lactobacillus reuteri ATCC 55730 in the treatment of patients with irritable bowel syndrome--a double blind, placebo-controlled, randomized study. Clin Nutr.

[23] Drakes M, Blanchard T, Czinn S (2004). Bacterial probiotic modulation of dendritic cells. Infect Immun.

[24] Rousseaux C, Thuru X, Gelot A (2007). Lactobacillus acidophilus modulates intestinal pain and induces opioid and cannabinoid receptors. Nat Med.

[25] Verdú EF, Bercik P, Verma-Gandhu M (2006). Specific probiotic therapy attenuates antibiotic induced visceral hypersensitivity in mice. Gut.

[26] Caballero-Franco C, Keller K, De Simone C, Chadee K (2007). The VSL#3 probiotic formula induces mucin gene expression and secretion in colonic epithelial cells. Am J Physiol Gastrointest Liver Physiol.

[27] Zeng J, Li YQ, Zuo XL, Zhen YB, Yang J, Liu CH (2008). Clinical trial: effect of active lactic acid bacteria on mucosal barrier function in patients with diarrhoea-predominant irritable bowel syndrome. Aliment Pharmacol Ther.

[28] Dunlop SP, Hebden J, Campbell E, Naesdal J, Olbe L, Perkins AC, Spiller RC (2006). Abnormal intestinal permeability in subgroups of diarrhea-predominant irritable bowel syndromes. Am J Gastroenterol.

[29] Marshall JK, Thabane M, Garg AX, Clark W, Meddings J, Collins SM (2004). Intestinal permeability in patients with irritable bowel syndrome after a waterborne outbreak of acute gastroenteritis in Walkerton, Ontario. Aliment Pharmacol Ther.

[30] Kok CR, Hutkins R (2018). Yogurt and other fermented foods as sources of health-promoting bacteria. Nutr Rev.

[31] Parvez S, Malik KA, Ah Kang S, Kim HY (2006). Probiotics and their fermented food products are beneficial for health. J Appl Microbiol.

[32] Fric P (2002). Probiotics in gastroenterology. Z Gastroenterol.

[33] de Vrese M, Schrezenmeir J (2008). Probiotics, prebiotics, and synbiotics. Adv Biochem Eng Biotechnol.

[34] Dimidi E, Christodoulides S, Scott SM, Whelan K (2017). Mechanisms of action of probiotics and the gastrointestinal microbiota on gut motility and constipation. Adv Nutr.

[35] Kim HJ, Vazquez Roque MI, Camilleri M (2005). A randomized controlled trial of a probiotic combination VSL# 3 and placebo in irritable bowel syndrome with bloating. Neurogastroenterol Motil.

[36] Dolin BJ (2009). Effects of a proprietary Bacillus coagulans preparation on symptoms of diarrhea-predominant irritable bowel syndrome. Methods Find Exp Clin Pharmacol.

[37] Sun YY, Li M, Li YY (2018). The effect of Clostridium butyricum on symptoms and fecal microbiota in diarrhea-dominant irritable bowel syndrome: a randomized, double-blind, placebo-controlled trial. Sci Rep.

[38] Satish Kumar L, Pugalenthi LS, Ahmad M, Reddy S, Barkhane Z, Elmadi J (2022). Probiotics in irritable bowel syndrome: a review of their therapeutic role. Cureus.

